# Dehydroepiandrosterone suppresses human colorectal cancer progression through ER stress-mediated autophagy and apoptosis in a p53-independent manner

**DOI:** 10.3389/fphar.2024.1464647

**Published:** 2024-10-02

**Authors:** Thi-Huong Nguyen, Huey-Jiun Ko, Po-Yu Tsai, Tai-Shan Cheng, Thu-Ha Tran, Ly Hien Doan, Michael Hsiao, Peter Mu-Hsin Chang, Hsiao-Sheng Liu, Yi-Ren Hong, Chi-Ying F. Huang

**Affiliations:** ^1^ Institute of Biopharmaceutical Sciences, National Yang Ming Chiao Tung University, Taipei, Taiwan; ^2^ Institute of Biotechnology and Food Technology, Thai Nguyen University of Agriculture and Forestry, Thai Nguyen, Vietnam; ^3^ Graduate Institute of Medicine, College of Medicine, Kaohsiung Medical University, Kaohsiung, Taiwan; ^4^ Department of Biochemistry, College of Medicine, Kaohsiung Medical University, Kaohsiung, Taiwan; ^5^ Institute of Biotechnology, Vietnam Academy of Science and Technology, Hanoi, Vietnam; ^6^ Genomics Research Center, Academia Sinica, Taipei, Taiwan; ^7^ Department of Oncology, Taipei Veterans General Hospital, Taipei, Taiwan; ^8^ Department of Medical Research, Kaohsiung Medical University Hospital, Kaohsiung Medical University, Kaohsiung, Taiwan; ^9^ Center for Cancer Research, College of Medicine, Kaohsiung Medical University, Kaohsiung, Taiwan

**Keywords:** Dehydroepiandrosterone (DHEA), colorectal cancer (CRC), ER stress induction, autophagy, apoptosis induction, p53 independence

## Abstract

Colorectal cancer (CRC) is one of the primary contributors to cancer-related fatalities, with up to 80% of advanced CRC cases exhibiting mutations in the p53 gene. Unfortunately, the development of new compounds targeting mutant p53 is quite limited. The anticancer effects of Dehydroepiandrosterone (DHEA) on various cancers have been reported. However, the suppressive effect of DHEA on CRC cells harboring wild-type or mutant p53 gene remains controversial. This study emphasized revealing the suppressive mechanism and the effect of DHEA on CRC cell tumorigenesis in the presence of wild-type or mutant p53 gene. We demonstrate that DHEA causes CRC cell death and cell cycle arrest in a dose and time-dependent manner. Notably, DHEA exhibits similar inhibitory effects on CRC cells regardless of the p53 gene status. Further study reveals that DHEA induces endoplasmic reticulum (ER) stress and triggers PERK/eIF2/ATF4/CHOP UPR signaling pathway to activate autophagy followed by apoptosis, which was confirmed by suppression of 4-phenylbutyric acid (an ER stress inhibitor) or knockdown either ATF4 or CHOP. DHEA-induced apoptosis was attenuated by silencing *ATG5* gene in either p53^+/+^ or p53^−/−^ CRC cells, indicating autophagy regulation of apoptosis. Furthermore, DHEA treatment accompanied by bafilomycin A1 (a blocker of autophagosome degradation) leads to the accumulation of ATF4, CHOP, DR5, and p21 levels in CRC cells, implying that the degradative autophagy machinery regulates these four molecules. Consistently, DHEA demonstrates its inhibitory effect by suppressing CRC tumor formation *in vivo*. Altogether, we provide compelling evidence that DHEA is a potential therapeutic candidate for CRC patient treatment regardless of the p53 status through ER stress-PERK-autophagy-apoptosis axis.

## Introduction

Colorectal cancer (CRC) has garnered worldwide attention due to the rising number of cases and cancer-related deaths. The progression of CRC frequently involves the increased activity of Kirsten rat sarcoma virus (*KRAS*) or/and beta-catenin pro-oncogenes, along with the loss of functions of tumor suppressors, including Adenomatous polyposis coli (*APC), SMAD4,* and p53*,* with mutation percentages ranging from 10% to 80% (Armaghany et al., 2012; Cancer Genome Atlas, 2012). In CRC, p53 mutation occurs at an approximate rate of 50%–60%, often following *APC* mutation at 80%. Most p53 mutations are missense types occurring at hotspots such as R273, R248, G245, R175, and R282. These mutations result in the loss of DNA binding sites, leading to impairment of the wild-type p53’s transcriptional function, which is crucial for inhibiting the cell cycle, repairing DNA, or inducing apoptosis in response to stress stimuli. Notably, p53 mutations are found in 80% of metastatic patients among advanced cases (Li et al., 2015; Nakayama and Oshima, 2019). The need for new therapeutic candidates that can address the challenges posed by these patients is urgent.

The importance of Endoplasmic Reticulum (ER) in maintaining cellular homeostasis of lipid biosynthesis, protein synthesis, and calcium storage has been documented. Various stress stimuli such as calcium and redox imbalances, hypoxia, acidosis, and exposure to chemical reagents, including natural compounds or marketing drugs, lead to the disruption of protein synthesis or protein folding and the subsequence of the increased amount of unfolded and misfolded proteins in the ER lumen (Schonthal, 2012). Under these stress conditions, Unfolded Protein Response (UPR) system, comprises three pathways: protein kinase R-like endoplasmic reticulum kinase (PERK), inositol-requiring kinase/endoribonuclease1 (IRE1), and activating transcription factor 6 (ATF6), is activated (Bhattarai et al., 2021). Among them, the activation of eukaryotic translation initiation factor 2A (eIF2α) phosphorylation by activated PERK, followed by induction of activating transcription factor 4 (ATF4) activity, is of great importance. ATF4 plays a crucial role in inducing gene expressions involved in adaptative processes, either by promoting survival or by stimulating pro-apoptotic genes, especially C/EBP homologous protein (CHOP) (Rozpedek et al., 2016; Zinszner et al., 1998). Upregulation of CHOP can trigger intrinsic and extrinsic apoptotic pathways through disruption of the mitochondrial membrane involved in the increase of BIM, BAX, and BAK or the upregulation of Death receptors (DR4 and DR5) signaling pathway (Hu et al., 2018). Moreover, increased ATF4 levels can partially restore wild-type p53-induced target genes, for example, p53 upregulated modulator of apoptosis (PUMA), NOXA, and DR5, in the cells with mutated p53 (Tian et al., 2021). Therefore, the activation of PERK/ATF4/CHOP axis represents one of potential mechanisms for targeting mutant p53 cells.

Autophagy is a degradative cellular process that helps to digest damaged organelles, unnecessary or non-functional proteins, and exogenous pathogens by delivering them to lysosomes for breakdown and recycling macromolecules to produce energy for cells (Kwon et al., 2023; Hale et al., 2013). In response to ER stress, the UPR system induces autophagy to eliminate unfolded and misfolded proteins, indicating a cytoprotective mechanism (Kwon et al., 2023; Yorimitsu et al., 2006). The activation of the PERK signaling pathway is crucial for inducing a series of autophagy-related genes, including *p62*/*SQSTM1*, *ATG*-related genes, and *Becn1* (B'Chir et al., 2013). Moreover, prolonged ER stress coupled with excessive autophagy can lead to cell death (Kwon et al., 2023; Rozpedek et al., 2016).

Dehydroepiandrosterone (DHEA) is a precursor hormone for androsterone and estrogen synthesis, primarily secreted from the adrenal cortex (Prough et al., 2016). DHEA can suppress various cancers through different mechanisms, including inhibition of the phosphatidylinositol-3-kinase (PI3K)/protein kinase B (AKT) pathway and induction of the autophagic cell death of liver cancer (Jiang et al., 2005; Vegliante et al., 2016). DHEA can also downregulate the WNT/beta-catenin pathway to inhibit cancer stem cells and sensitize irinotecan in the head and neck cancer (Li et al., 2022), as well as upregulate the p53 pathway in the breast cancer (Yang et al., 2016). DHEA has also been demonstrated to act as a non-competitive glucose-6-phosphate Dehydrogenase (G6PD) enzymatic inhibitor (Marks and Banks, 1960) or an inhibitor of cholesterol and isoprenoid synthesis through endogenous mevalonate deletion in CRC (Schulz and Nyce, 1991). In addition, G6PD activity can also be inhibited by wild-type p53 through the prevention of active dimer formation (Jiang et al., 2011). However, the relationship between p53 status and the anti-cancer effects of DHEA on CRC remains unclear.

In this study, we aimed to elucidate the relationship between p53 status and the inhibitory effect of DHEA *in vitro* and *in vivo*, utilizing various CRC cell lines harboring different p53 statuses. Our findings offer a promising foundation for future clinical application of CRC patients with different p53 status.

## Materials and methods

### Cell lines and cell culture

Most of the cell lines used in this study were colorectal adenocarcinoma cells. RKO (wild-type p53) and HT29 (mutant p53, p.R273H) were generous gifts of Professor Michael Hsiao (Genomics research center, Academia Sinica, Taiwan). RKO cells were maintained in Minimum Essential Medium (MEM). HT29 cells were cultured in Mccoy’s 5A medium. HCT116 (with p53 ^+/+^ - wild-type or WT, and p53^−/−^ -null) cells were a kind gift from Professor Won-Jing Wang (Institute of Biochemistry and molecular Biology, National Yang Ming Chiao Tung University, Taipei 112, Taiwan), cultured in Dulbecco’s Modified Eagle Medium (DMEM). LS1034 cells were obtained from the Bioresource Collection and Research center (BCRC), Taiwan, and were cultured in Roswell Park Memorial Institute (RPMI 1640) medium. Complete medium supplemented with 10% fetal bovine serum (FBS), 1% penicillin, 1% streptomycin, and 1% sodium pyruvate (for RKO cells). All media and reagents were purchased from Gibco, Thermo Fisher Scientific, Grand Island, NY, United States. All cell lines were maintained in a 5% CO_2_ incubator at 37°C. Cell passaging was performed every 3 days through trypsinization using 1X trypsin from Gibco, Thermo Fisher Scientific, Grand Island, NY, United States. Trans-Dehydroandrosterone (DHEA) was purchased from Sigma-Aldrich in China, with Lot#MKCH2793, and it was dissolved in dimethyl sulfoxide (DMSO) to prepare a stock solution at a concentration of 200 mM.

### Sulforhodamine B (SRB) assay

Cells (3 × 10^3^ cells) were seeded into each well of a 96-well plate, and grew overnight at 37°C in an atmosphere containing 5% CO_2._ Subsequently, the cells underwent treatment with a range of DHEA concentrations spanning from 0 to 400 μM for 24, 48, and 72 h. After harvesting, the cells were fixed with 10% trichloroacetic acid (TCA), obtained from Sigma (cat. SI-T6399-250G), and left to incubate overnight at 4°C. Following this, the cells underwent a water rinse and were left to air dry at room temperature (RT). The next step involved staining the cells with Sulforhodamine B (SRB) (Sigma, cat. S1402) at a concentration of 0.057% for 1 h at RT. After the staining period, the cells were rinsed with 1% acetic acid (J.T Baker, cat. JT-9508-03). Subsequently, the stained cells were allowed to air dry at RT before being completely dissolved in 200 μL of 10 mM Tris-base (Amresco, cat. CPT-0826). The colorimetric intensity of the SRB dye was assessed at 510 nm using Elisa reader (TECAN Infinite 200 Pro). Data were presented as percentage of inhibition ± Standard Error of mean (SEM) obtained from three different experiments.

### CCK-8 assay

Both HCT116 p53^+/+^ and HCT116 p53^−/−^ (3,000 cells per well) were plated into a 96-well plate and fully attached to the plates overnight. Subsequently, DHEA was administered to the cells at concentrations of 0, 50, 100, 200, and 250 μM for treatment durations of 0, 24, 48, and 72 h. At each time point, cells were incubated for 3 h with cell counting kit-8 (CCK-8) reagent (TargetMol, cat no: C0005) at a one-tenth ratio, maintaining the incubation at 37°C in an atmosphere with 5% CO_2_. Cell proliferation was determined by measuring OD value at 450 nm using an Elisa reader equipped with Gen5 software. The data were presented as OD values at various time points during the treatment period with ± Standard Error of the mean (SEM) from three different experiments.

### Cell cycle assay

5 × 10^5^ cells were plated into 6-cm dishes. Following overnight incubation at 37°C with 5% CO_2_, the cells were incubated with various concentrations of DHEA, including 0, 100, and 200 μM for 48 h. After 48 h treatment, the cells were rinsed twice with cold PBS 1X and then scraped into 15 mL centrifuge tubes. Subsequently, the suspended cells were centrifuged at 1 500 rpm for 5 min to remove the supernatant. The cell pellets were separated by 1 mL of cold PBS 1X and fixed by adding methanol at a 1:2 ratio of PBS to methanol, followed by an overnight incubation at 4°C. The fixed cells were then collected by centrifugation at 1 500 rpm for 10 min to remove the fixing solution. After that 1 mL of room temperature PBS was added to resuspend cell pellets, and the suspension was transferred to 1.5 mL tubes. Repeat the centrifugation step to collect cell pellets. Subsequently, the cells were incubated with propidium iodide (PI) (Sigma-Aldrich, CAS number 25535-16-4, branch in Taiwan) working solution, which included PI 20 μg/mL and RNase A 2.5 μg/mL calculated at final concentration). Staining was carried out for 30 min at 37°C with protection from light. The stained cells were then assessed using Attune NxT flow cytometer from Thermo Fisher Scientific. Data was collected for a minimum of 10 000 events based on PI intensity. The percentage of cell population in each phase was calculated by Attune software. The data was obtained from three independent experiments.

### Colony formation assay

Cells (1 × 10^3^/well) were plated into a 6-well plate, and then the cell grew overnight in a 5% CO_2-_supplying incubator at 37°C. The cells were then treated with a range of DHEA concentrations, including 0, 25, 50, 100, 150, and 200 μM, for a duration of 12 days. Every 3 days, cells were refreshed with a medium containing the drug was replenished. After a 12-day treatment period, the cells underwent washing and fixation with 4% paraformaldehyde for 30 min at RT. Subsequently, paraformaldehyde was removed with water, and 0.5% crystal violet was applied to stain the cells for another 30 min at RT. Colony numbers were counted by ImageJ software. The percentage of colony inhibition was calculated by normalizing the treatment group to the control group. The data were presented as the percentage of colony inhibition and were derived from three independent experiments.

### Apoptotic assay

Cells were plated into 6-cm dishes with a density of 5 × 10^5^ cells and incubated in the incubator with 5% CO_2_ and at 37°C. After plating for 24 h, cells were treated with DHEA at 0, 100, and 200 μM for 48 h. Apoptotic cells were stained by either/both Annexin V or/and propidium iodide (PI) dye following the manufacturer’s instruction (CF^®^488A Annexin V and PI apoptosis kit, Cat No. 30061, Biotium). Briefly, pelleted cells obtained by centrifugation were resuspended and incubated with 100 μL of 1 × Annexin V buffer containing 5 μL of CF^®^ 488A Annexin V and 2 μL of PI working solution for 30 min at RT with light protection. Then stained cells were determined by BD FASCLyric™ flow cytometer, and results were analyzed by FlowJo V.10 software for a percentage of each population of PI-stained cells, Annexin V-stained cells, or both Annexin V and PI-stained cells. Here, apoptotic cells were calculated from cells stained with Annexin V only and both Annexin V and PI. Data were collected from three independent experiments.

### Lentiviral infection

Cell density with 2 × 10^5^ cells was plated in a 6-well plate and, after 14 h of incubation at 37°C, supplied 5% CO_2_. Lentiviruses containing the plasmids (Supplementary Table S1) purchased from RNAicore, Academic Sinica, Taiwan, were infected into the cells for an additional 12 h, using a Multiplicity of Infection (MOI) of 2. Infected cells were washed twice with PBS 1 × to remove excessive lentivirus. Fresh medium containing puromycin at a final concentration of 2 μg/mL was added to select successfully infected cells over a 48-h period. After this selection, death cells were washed out with 1X PBS. Subsequently, the cells were allowed to recover in a fresh medium without puromycin overnight. The lentiviral-infected cells were then sub-cultured to amplify and assess the knockdown efficiency by Western Blots before proceeding with further experiments. Following subculturing, the cells were consistently maintained in a medium containing puromycin at a concentration of 2 μg/mL.

### Western blot analysis

Cells (2 × 10^6^) were initially plated in 10-cm dishes and were then subjected to a single treatment with DHEA at doses of 0, 100, 200 μM. Additionally, cotreatment experiments were conducted using either Bafilomycin A1 (BafA1) at a concentration of 100 nM or 4-phenylbutyric acid (4-PBA) at 1 mM (MedChemExpress, cat. No: HYA0281), all for a duration of 48 h. Cell lysates were collected after cell pellets were lysed using RIPA lysis buffer, which was purchased from EMD Millipore Billerica, MA, United States) in a 10 × concentration, then added protease inhibitor 5 μL/mL and phosphatase inhibitors 10 μL/mL. The protein amount was identified using a Bio-Rad protein assay kit (Bio-Rad Laboratories, Hercules, CA, United States). Investigated proteins were separated by SDS-PAGE gels with the percentage of concentrations ranging from 8% to 12%, then transferred to PVDF-membranes. Non-specific proteins were blocked by 5% skim milk for 1 h at RT. Membranes were rinsed three times with 1X TBST buffer. After that, the membranes were incubated with primary antibodies (Supplementary Table S2) at 4°C overnight on a shaker. They were then incubated with either mouse or rabbit horseradish peroxidase (HRP) – conjugated secondary antibodies for 1 h at RT. Then, after another round of washing three times with 1 × TBST buffer, proteins were incubated with sensitive ECL substrates and captured by Multigel 21. GAPDH and α-Tubulin served as internal controls. Band intensities were quantified by Mutigaugel 3.0 software.

### Xenograft mouse model for CRC tumor formation

The protocol of animal experiments was conducted following the instructions of the Care and Use of Laboratory Animals of the National Institutes of Health (NIH) and approved by the Institutional Animal Care and Use Committee of Academia Sinica (Taipei, Taiwan: IACUC no: AS15-06-833). Briefly, male 6-week-old NOD/SCID mice were subcutaneously inoculated with HT29 cells (5 × 10^6^). Ten mice were divided into DMSO control and DHEA treatment two groups with five mice in each group. After the first week of inoculation, tumor-bearing mice were administered either DMSO or DHEA (16 mg/kg) for 3 weeks via intraperitoneal injection 3 times/week and sacrificed at the fourth week. Body weight was measured once a week before the drug injection. Tumor weight was measured after scarification.

### Statistical analysis

The results are presented as the mean ± Standard Error of Mean (SEM). Statistical significance was obtained by comparing control and treatment groups using a Student’s t-test, one- or two-way ANOVA with Turkey’s multiple comparison test. In which non-significant (ns) corresponds to p-value > 0.05; * or ^*#*^ p < 0.05; ** or ^*##*^ p < 0.01; *** or ^*###*^ p < 0.001; **** or ^*####*^ *p < 0.0001*. Statistical comparisons and plotted figures were conducted by GraphPad Prism 9.0 software (DMCA Compliance Agent, GraphPad Software, BOSTON, USA).

## Results

### DHEA suppresses the growth and colony formation of human CRC cells regardless of p53 status

**FIGURE 1:**
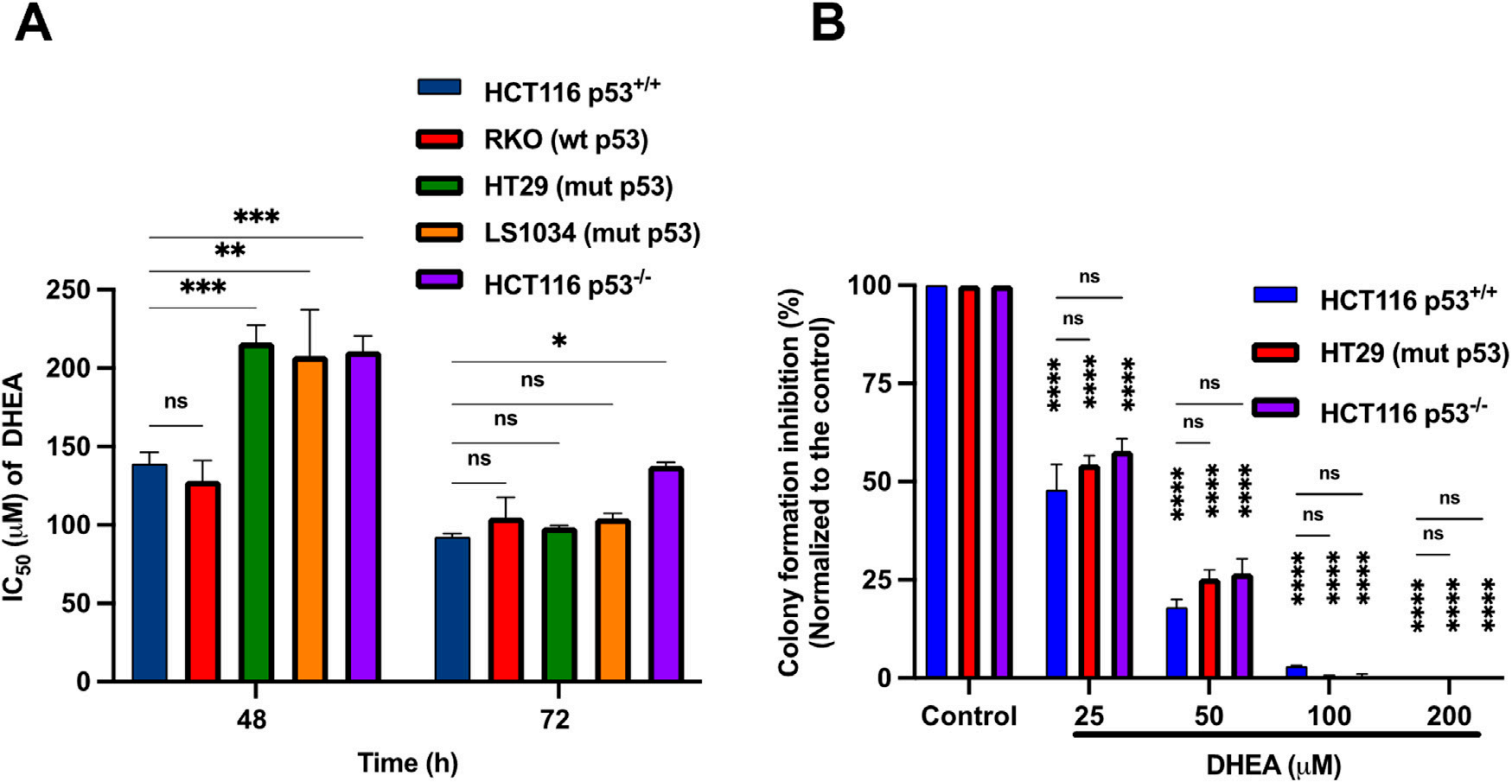
DHEA suppresses the growth and colony formation of human CRC cells regardless of p53 status. **(A)** IC_50_ values of DHEA of five human CRC cell lines, including wild-type p53 (HCT116 p53^+/+^ and RKO), mutant p53 (LS1034 and HT29), and null p53 (HCT116 p53^−/−^) cells were determined after 48 and 72 h of DHEA treatment. The IC_50_ values were determined by measuring the optical density (OD) at wavelength 510 nm using SRB assay. Statistical analysis was conducted by comparing with HCT116 p53^+/+^; ns: non-significance with *p-value* > 0.05, *p < 0.05; **p < 0.01; ***p < 0.001; ****p < 0.0001. **(B)** The percentage of inhibition of colony formation by DHEA, ranging from 0 to 200 μM, was determined after 12 days of treatment on HCT116 p53^+/+^, HT29, and HCT116 p53^−/−^ cells. Statistical analysis was conducted to compare the effect of DHEA between treatment groups and the untreated group of HCT116 p53^+/+^, HT29, and HCT116 p53^−/−^ cells and between 3 cells. Statistical data were obtained from three independent experiments with ns: non-significant with *p-value* > 0.05, ****p < 0.0001; Error bar is Standard Error of Mean (SEM).

Despite the anticancer effects of DHEA on CRC have been reported (Jiang et al., 2005; Schulz et al., 1992; Schulz and Nyce, 1991), the role of p53 in DHEA suppression of CRC has not been explored. Herein, five CRC cells harboring wild-type p53 (HCT116^+/+^ or HCT116 p53^+/+^ and RKO), mutant p53 (HT29 and LS1034), or null p53 (HCT116^−/−^ or HCT116 p53^−/−^) were analyzed. From previous studies, the doses of DHEA have shown anti-cancer effects ranging from 50 to 400 μM *in vitro* treatment, observed in various types of cancer cells (Giron et al., 2009; Li et al., 2022; Ortega-Calderon and Lopez-Marure, 2014; Schulz et al., 1992; Schulz and Nyce, 1991; Siegel et al., 2023; Vegliante et al., 2016; Zhang et al., 2024). Thus, in this study, *in vitro* CRC cancer cell lines were treated with different concentrations of DHEA (0–400 μM) for varying durations. The growth suppressive effect of DHEA was then assessed using the SRB assay. Our data showed that IC_50_ values of the two wildtype p53 cell lines were significantly lower than those of the three p53 mutant cell lines at 48 h post-DHEA treatment (p.t.). Intriguingly, the IC50 values of all 5 CRC cell lines decreased and showed no significant difference at 72 h p.t. (Figure 1A; Supplementary Table S3). Moreover, DHEA dose-dependently suppressed the growth of the five CRC cell lines at 48 h and 72 h p.t. (Supplementary Figure S1A). Our data imply that DHEA showed a dose-dependent suppression of CRC cells regardless of the status of p53 at 72 h p.t. Furthermore, the inhibitory effect of DHEA on the anchorage-independent colony formation of three CRC cell lines (HCT116^+/+^, HT29, and HCT116^−/−^) was assessed. DHEA effectively suppressed the number of colony formation of 3 cells in a dosage-dependent manner at day 12 p.t. (Figure 1B; Supplementary Figure S1B). Taken together, DHEA suppresses CRC cells regardless of p53 status in a dose- and time-dependent manner.

### DHEA suppresses CRC cell viability by increasing G1 phase arrest and apoptosis in a p53-independent manner

**FIGURE 2:**
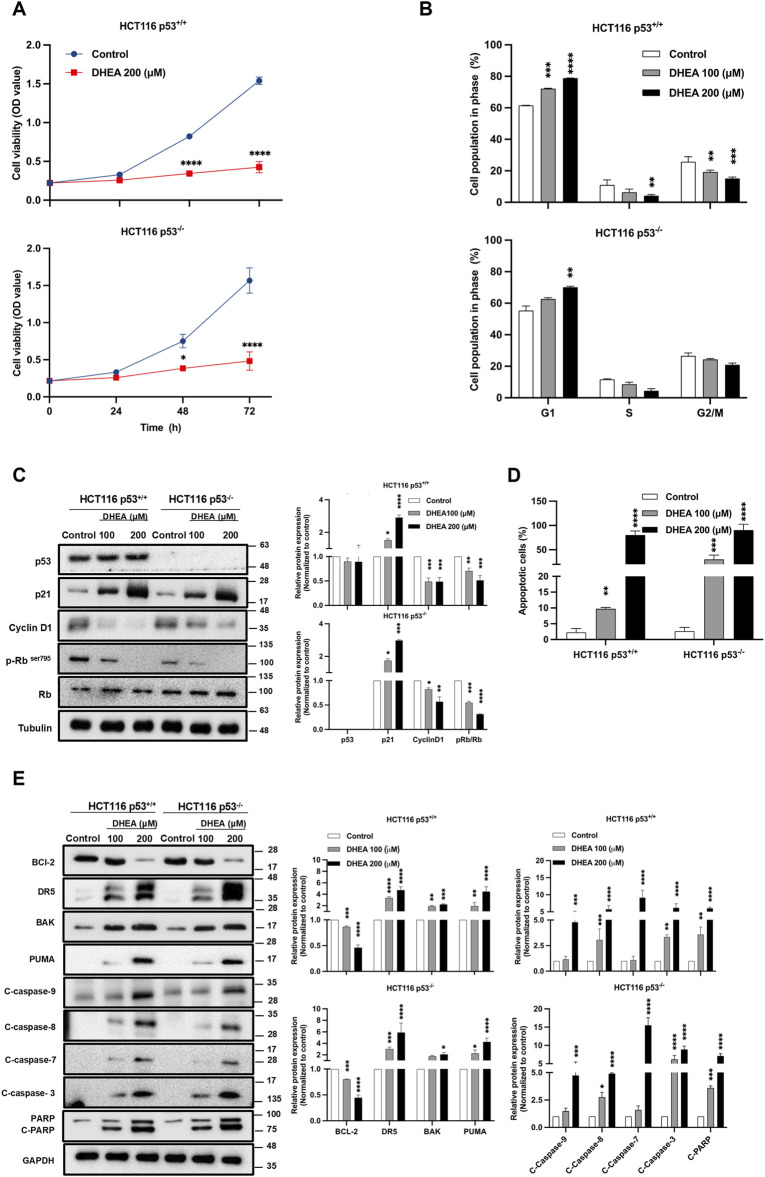
DHEA suppresses CRC cell viability by increasing G1 phase arrest and apoptosis in a p53-independent manner. **(A)** Cell growth of p53^+/+^ and p53^−/−^ HCT116 cells were assessed after treatment with a concentration of DHEA (200 µM) for 0, 24, 48, and 72 h using CCK-8 assay at wavelength 450 nm. Statistical analysis was performed across three independent experiments to compare the inhibitory effect of DHEA on cell viability between the control group (treated with DMSO) and the DHEA treatment group at each time-point with (*p < 0.05; ****p < 0.0001). **(B)** Cell cycle arrest at G1 phase of p53^+/+^ and p53^−/−^ HCT116 cells was determined by propidium iodide (PI) staining and analyzed by flow cytometry after 48 h of DHEA treatment at 0, 100, and 200 µM. Statistical analysis was conducted to compare the percentage of cell population at each phase of the cell cycle to its respective control group (treated with DMSO) with **p < 0.01; ***p < 0.001; ****p < 0.0001. Western blots result of specific proteins after DHEA treatment at 0, 100, and 200 µM for 48 h in both p53^+/+^ and p53^−/−^ HCT116 cells, **(C)** showed the protein expression of cell cycle markers at the G1 checkpoint and **(E)** the expression of apoptotic markers. Western Blotting images were captured by Multigel 21. The intensity of bands was quantified by MultiGauge 3.0 software. Tubulin and GAPDH were used as internal controls. Statistical quantified data were obtained by comparing the control and DHEA-treated groups with *p < 0.05; **p < 0.01; ***p < 0.001; ****p < 0.0001. **(D)** The percentage of apoptotic cells stained by Annexin V or/and PI post DHEA treatment at 48 h in two HCT116 cells was determined by flow cytometry and analyzed by FlowJo V.10 software. Statistical analysis compared the control and treatment groups with **p < 0.01; ***p < 0.001; ****p < 0.0001.

The following studies used HCT116^+/+^ and HCT116^−/−^ cell lines to clarify further whether p53 participates in DHEA suppression of CRC tumorigenesis *in vitro*. The CCK-8 assay was conducted to monitor the viability of the cells from 0 to 72 h post-DHEA treatment (p.t.). The data showed that DHEA significantly suppressed the viability of HCT116^+/+^ and HCT116^−/−^ cells in a time-dependent fashion. Notably, DHEA showed similar inhibitory effects on these 2 cell lines (Figure 2A). Cell cycle analysis was conducted to clarify how DHEA suppresses cell viability. Our data showed that DHEA leads to significant cell accumulation at the G1 phase accompanied by a decrease at the S and G2/M phases in both HCT116^+/+^ and HCT116^−/−^ cells while the concentration of DHEA was increased 48 h p.t. (Figure 2B; Supplementary Figure S2B). To clarify the molecules participating in DHEA-related cell cycle arrest at the G1 phase, cell cycle-related proteins were investigated by Western blot analysis. After DHEA treatment, cyclin D1 and phospho-retinoblastoma at ser 795 site (p-Rb (ser795) levels decreased with the increase of p21 protein dose-dependent with or without the p53 gene (Figure 2C).

DHEA suppressed CRC cell viability and caused cell cycle arrest, indicating that the treated cells may undergo apoptosis. It was observed that DHEA dose-dependently induced cell apoptosis of HCT116^+/+^ and HCT116^−/−^ cells at 48 h p.t. (Figure 2D; Supplementary Figure S2B). Consistently, DHEA dose-dependently increased the cleavage of caspase 9, 8, 7, 3, and PARP, accompanied by the increase of pro-apoptotic markers, including DR5, BAK, and PUMA, as well as a notable decrease of the anti-apoptotic marker BCL-2 in HCT116^+/+^ and HCT116^−/−^ cells (Figure 2E). Above data implies that DHEA treatment induces apoptosis of CRC cells in a p53-independent fashion. In summary, DHEA exerted its anti-cancer activity on CRC cells by blocking the cell cycle at the G1 phase, followed by induction of apoptosis in a p53 dispensable manner.

### DHEA induces ER stress through PERK/eIF2/ATF4/CHOP UPR signaling pathway in a p53-independent manner

**FIGURE 3:**
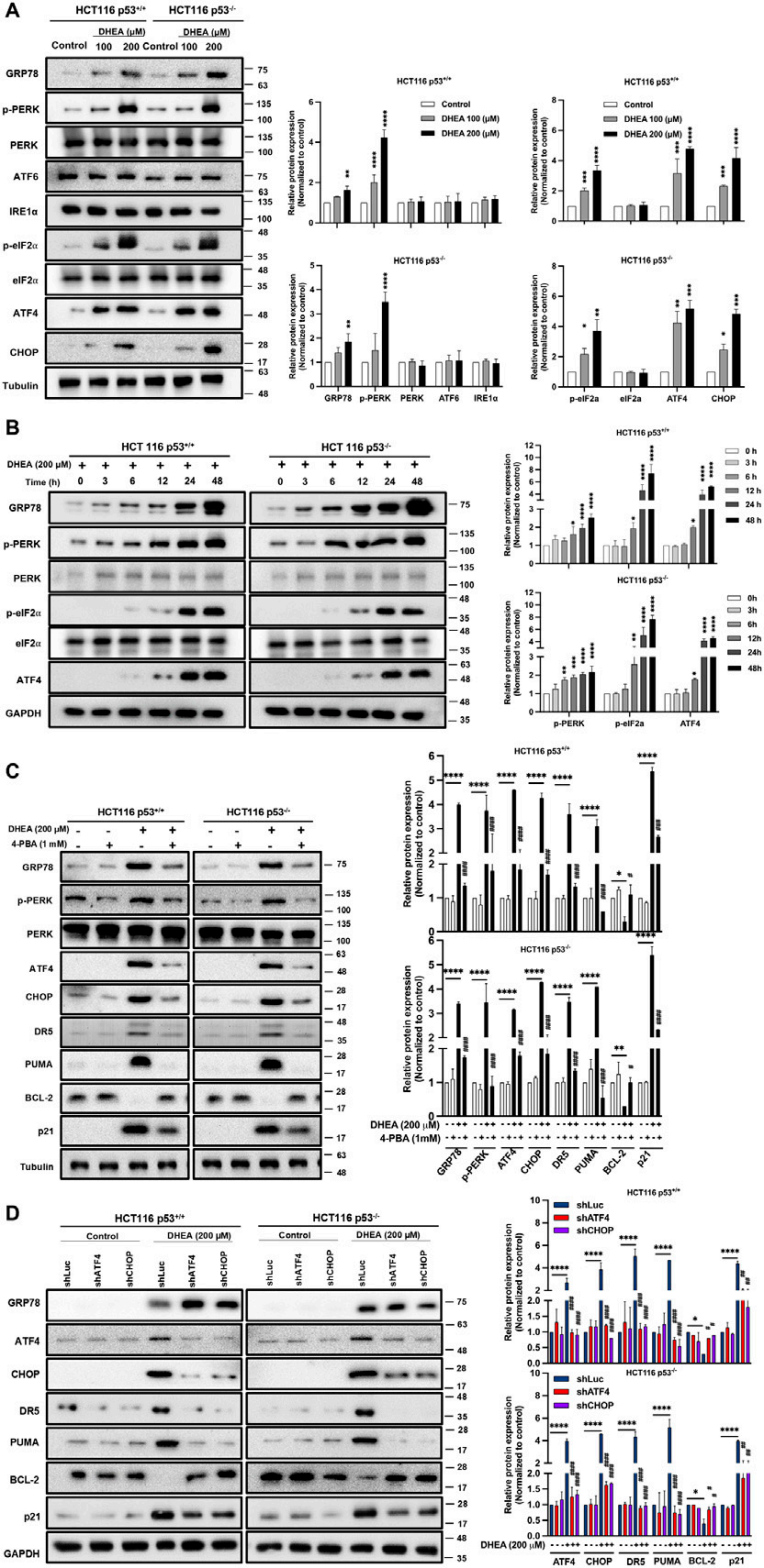
DHEA induces ER stress through PERK/eIF2/ATF4/CHOP UPR signaling pathway in a p53-independent manner. **(A)** Both p53^+/+^ and p53^−/−^ HCT116 cells were treated with various doses of DHEA (0, 100, and 200 µM) for 48 h. Cell lysates were collected to assess the protein expression associated with the ER stress using specific antibodies through Western blot assay. **(B)** The response of ER stress-related proteins to 200 µM DHEA treatment was monitored from 0 h to 48 h for p53^+/+^ and p53^−/−^ HCT116 cells. **(C)** p53^+/+^ and p53^−/−^ HCT116 cells were treated with 4-PBA (an ER stress inhibitor) in the presence or absence of 200 µM DHEA for 48 h. GRP78 (BiP, ER stress marker), PERK UPR pathway (PERK, p-PERK, ATF4, and CHOP), and its downstream target genes (DR5, PUMA, BCL-2, and p21) were examined using specific antibodies. **(D)** Two HCT116 cell lines were infected with lentiviral shRNA of ATF4 and CHOP with or without DHEA (200 µM) for 48 h. The marker proteins of ER stress, the PERK pathway, and downstream target genes were assessed by Western blot assay. GAPDH and tubulin were utilized as internal controls. Statistical results were obtained from three independent experiments by comparing the control group and the DHEA treatment group with *p < 0.05; **p < 0.01; ***p < 0.001; ****p < 0.0001; and by comparing the control group with gene silencing group or 4-PBA group under DHEA treatment with ^#^ *p* < 0.05; ^##^ *p* < 0.01; ^####^ *p* < 0.0001.

Others have reported that ATF4, a transcription factor downstream of ER stress signaling pathways, can induce p21 expression (Inoue et al., 2017). It can also upregulate pro-apoptotic protein expression, including CHOP, PUMA, and DR5, independent of p53 regulation (Tian et al., 2021). DHEA treatment might also lead to the accumulation of reactive oxidative stress (ROS) by inhibiting G6PD enzyme activity (Marks and Banks, 1960) or by inhibiting protein isoprenylation to increase non-isoprenylated proteins (Schulz and Nyce, 1991), which can trigger ER stress (Mele et al., 2018; Nam et al., 2021). Based on these findings, we hypothesize that DHEA may induce ER stress and trigger apoptotic cell death. Accordingly, we clarified the level of ER stress and downstream three unfolded protein response (UPR) pathways in DHEA-treated CRC cells using the markers GRP78 (BiP), ATF6, IRE1α, and PERK representing ER stress and three UPR pathways. Our data showed that DHEA dose-dependently increased protein levels of ER stress marker GRP78 and downstream PERK UPR pathway markers, including phosphorylated PERK, phosphorylated eIF2α, and ATF4. In contrast, DHEA could not increase the levels of ATF6 and IRE1α UPR pathways at 48 h p.t. (Figure 3A). In addition, DHEA showed time-dependent upregulation of GRP78, phosphorylated PERK, phosphorylated eIF2α, as well as ATF4 (Figure 3B). Moreover, DHEA had no effect on total protein levels of PERK and eIF2α as well. Altogether, our findings clearly demonstrate that DHEA-triggered ER stress mainly activates PERK/eIF2/ATF4 signaling pathway to suppress CRC cell proliferation demonstrated in p53^+/+^ and p53^−/−^ CRC cells. To validate our speculation, two HCT116 cell lines were treated with the ER stress inhibitor 4-phenylbutyric acid (4-PBA, 1 mM) in the presence or absence of DHEA (200 μM) for 48 h. Consistent with our previous findings, DHEA alone induced ER stress through the PERK signaling pathway, as evidenced by a significant increase of GRP78, p-PERK, ATF4, and CHOP expression, and coincided with increased downstream proteins, including DR5, PUMA, p21, and downregulated of BCL-2. However, the above DHEA effect was evidently mitigated by 4-PBA accompanied by the reduction of GRP78, p-PERK, ATF4, CHOP, DR5, PUMA, and p21 protein levels, while the increase of BCL-2 (Figure 3C). To confirm the role of the PERK pathway in regulating wild-type p53 targets, we silenced ATF4 and CHOP, two important transcriptional factors, using shRNA lentiviral systems. As shown in Figure 3D, silencing either ATF4 or CHOP (encoded by *DDIT3* gene) led to attenuation of the effect of DHEA on downstream genes, including DR5, PUMA, p21, and BCL-2 but not in the upstream of ATF4 and CHOP. These data imply that DHEA might restore wild-type p53 target genes through the PERK pathway.

Similarly, DHEA also induced ATF4, CHOP, and p21 protein expression in two other CRC cell lines, including HT29 and LS1034 harboring various mutant p53 genes (Supplementary Figure S3). Taken altogether, our data clearly demonstrate that DHEA treatment of CRC cells induces ER stress and downstream PERK/eIF2α/ATF4/CHOP axis to execute its tumorigenesis in a p53-independent manner.

### DHEA-triggered PERK/ATF4/CHOP UPR pathway induces autophagic activity in a p53-independent manner

**FIGURE 4:**
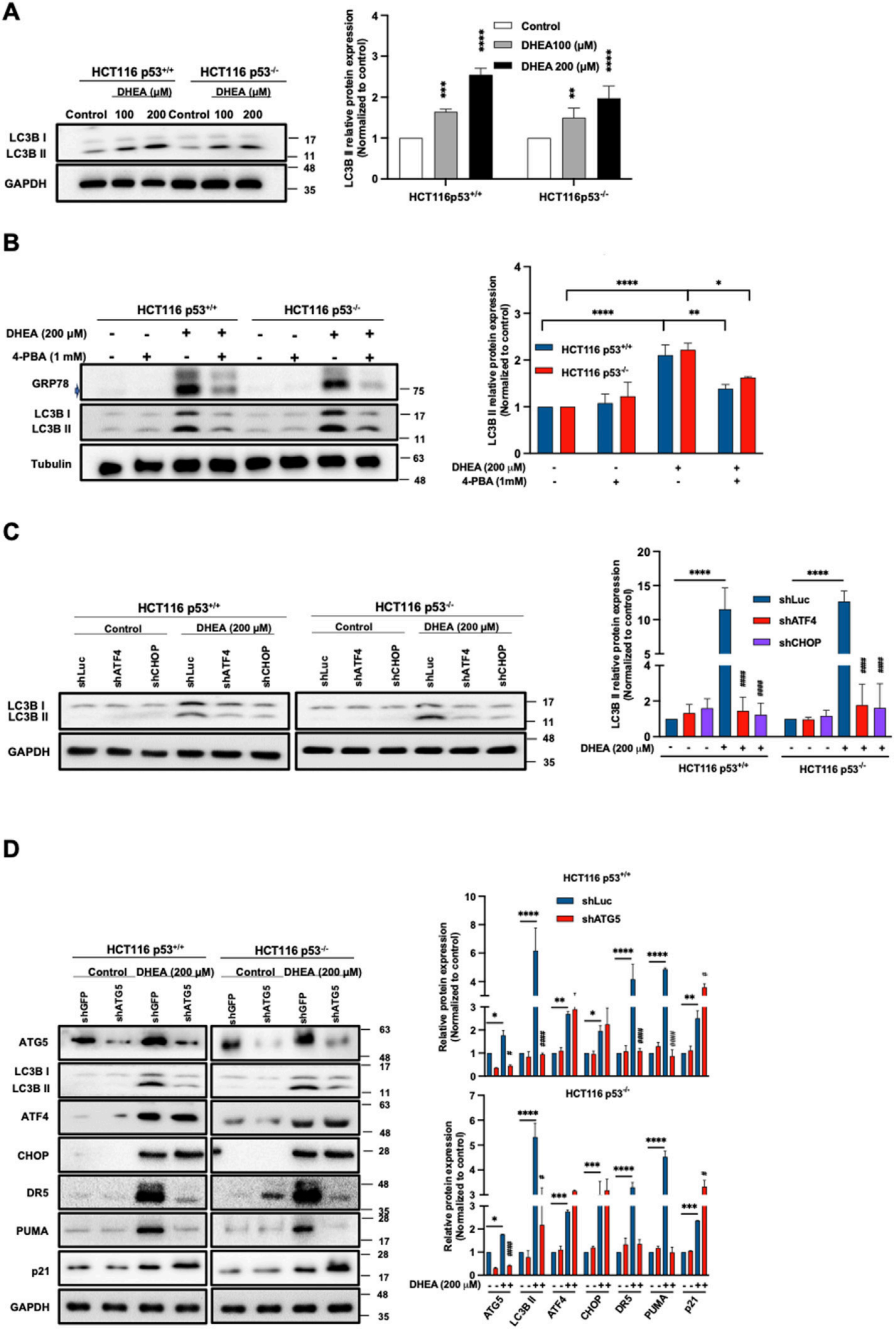
DHEA-triggered PERK/ATF4/CHOP UPR pathway induces autophagic activity in a p53-independent manner. **(A)** HCT116 p53^+/+^ and p53^−/−^ cells were treated with DHEA at concentrations of 0, 100, and 200 µM for 48 h LC3B II protein (representing autophagy progression) was evaluated using specific antibodies by Western blot assay. **(B)** GRP78 and LC3B II protein levels after 4-PBA treatment with or without DHEA (200 µM) for 48 h in p53^+/+^ and p53^−/−^ HCT116 cells. **(C)** Lentiviral shRNA targeting *ATF4* or *DDIT3* (encoded CHOP protein) genes were used in p53^+/+^ and p53^−/−^ HCT116 cells. A control group was treated with a lentiviral shLuciferase system (shLuc). Subsequently, the treated cells were exposed to DMSO (control group) and DHEA (200 µM) for 48 h, and LC3B protein level was assessed using specific antibodies by Western blotting. **(D)** The *ATG5* gene of p53^+/+^ and p53^−/−^ HCT116 cells were silenced by lentiviral shRNA. Lentiviral shRNA containing Green fluorescent protein gene (shGFP) was used as a vehicle. These lentiviral shRNA-treated cells were treated with DMSO (control group) or DHEA (200 µM) for 48 h. LC3B, ATF4, CHOP, DR5, PUMA, and p21 proteins were determined by Western blotting using specific antibodies. Tubulin and GAPDH served as internal controls. Statistical data were from three independent experiments comparing the control and DHEA treatment groups. *p < 0.05; **p < 0.01; ***p < 0.001; ****p < 0.0001; and by comparing the control group with gene silencing group under DHEA treatment with ^#^ *p* < 0.05; ^##^ *p* < 0.01; ^####^ *p* < 0.0001.

Autophagy activation triggered by ER stress may maintain homeostasis of the ER lumen by eliminating the unfolded and/or misfolded proteins during protein synthesis (Bhattarai et al., 2021; Ogata et al., 2006). Autophagy dysfunction has been reported to cause cell death (Xi et al., 2022). To clarify whether DHEA-induced ER stress could affect autophagy, the protein level of LC3B II (a marker of autophagosome formation) was evaluated in HCT116 p53^+/+^ and HCT116 p53^−/−^ cells. Our data showed that DHEA significantly induced LC3B II protein expression, and this effect was further enhanced by increased doses (Figure 4A). Similarly, DHEA induced autophagy in the other two CRC cell lines, HT29 and LS1034, harboring different mutant p53 genes (Supplementary Figure S4). To clarify the relationship between DHEA-triggered ER stress and autophagy induction, we blocked ER stress by the pharmacological inhibitor 4-PBA, downstream PERK UPR pathway ATF4, and CHOP gene expression by shRNA genetic silencing, as well as autophagy function by genetic silencing Autophagy related 5 (*ATG5)* gene of the CRC cells for 48 h in the presence or absence of DHEA (200 μM). Our data showed that 4-PBA effectively attenuated DHEA-induced ER stress accompanied by decreased levels of LC3B II compared to DHEA treatment alone in either p53^+/+^ or p53^−/−^ HCT116 cells (Figure 4B). Similarly, by silencing either ATF4 or CHOP gene, the protein level autophagy LC3B II was significantly decreased compared to the parental group after DHEA treatment (Figure 4C). Moreover, knockdown *ATG5* gene, an essential gene for autophagosome formation (B'Chir et al., 2013), led to significant suppression of LC3B II in CRC cells under DHEA treatment. Intriguingly, silencing *ATG5* gene significantly suppressed DR5 and PUMA without affecting DHEA-induced ATF4, CHOP, and p21 levels in two HCT116 cell lines (Figure 4D). Altogether, our findings imply that DHEA-triggered ER stress-ATF4-CHOP pathway regulates autophagic activity, and autophagy regulates DR5 and PUMA but not p21 upregulation.

### DHEA-induced autophagy degradation machinery leads to cell apoptosis in a p53-independent manner

**FIGURE 5:**
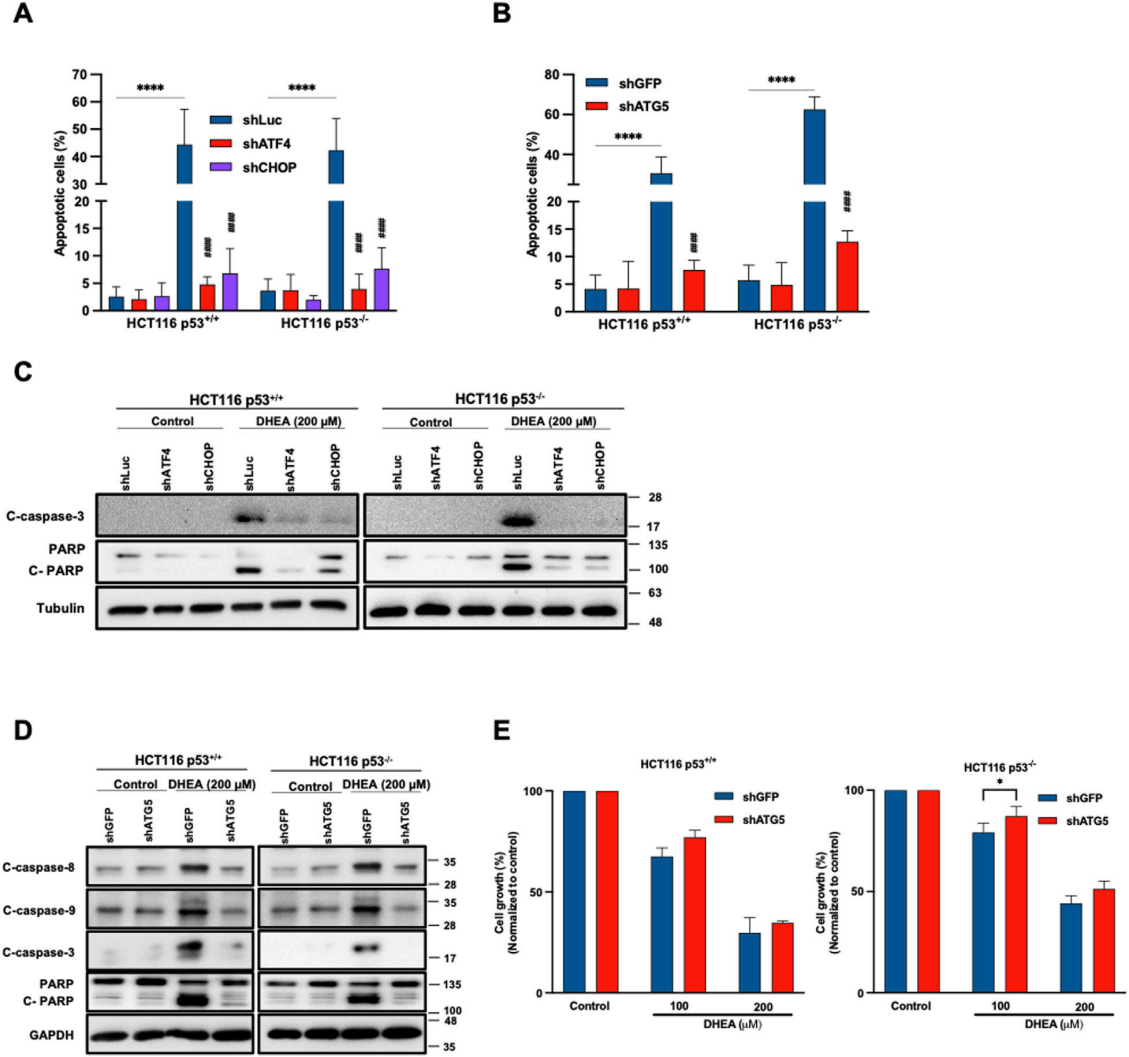
DHEA-induced apoptosis mediates autophagy in a p53-independent manner. HCT116 p53^+/+^ and p53^−/−^ cells were treated with lentiviral shRNA of *ATF4*, CHOP, *ATG5*, or Luciferase and GFP (vehicle) with or without DHEA (200 µM) for 48 h **(A, B)** The percentage of apoptotic cells was determined by staining Annexin V and PI dyes after DHEA treatment at the indicated time, analyzed by flow cytometry for shATF4, shCHOP, and shATG5, respectively. *p < 0.05; **p < 0.01; ***p < 0.001; ****p < 0.0001; and by comparing the control group with the gene silencing group under DHEA treatment with ^####^ *p* < 0.0001. **(C, D)** Western blot analysis was conducted to evaluate the levels of apoptosis proteins caspase 8, 9, and 3, and PARP using specific antibodies. GAPDH and tubulin were used as the internal controls. **(E)** The percentage of cell growth of two parental and *ATG5*-silenced HCT116 cells after DHEA treatment at 100 and 200 µM for 72 h was determined by SRB assay. The statistical result was obtained by comparing the control and DHEA treatment groups with *p < 0.05.

Lentiviral shRNA was used to silence *ATG5* gene to clarify whether autophagy might participate in DHEA-induced apoptosis. Additionally, silenced *ATF4* and *DDIT3* CHOP) genes were silenced to verify the relationship between PERK UPR and autophagy in inducing apoptosis. Our data showed that DHEA significantly induced apoptosis of parental cells, which was remarkably attenuated by knocking down *ATF4*, *DDIT3* (CHOP) (Figure 5A; Supplementary Figure S5A), or *ATG5* (Figure 5B; Supplementary Figure S5B). Consistent with inducing apoptotic cells, the levels of cleaved forms of caspase 3 and PARP (representing apoptosis) were evidently decreased in *ATF4*, *DDIT3* (CHOP), or *ATG5-*silenced HCT116 cell lines in the presence of DHEA compared to the parental groups (Figures 5C,D). Notably, the silencing of *ATG5* also reduced protein levels of cleaved caspase 8 and 9 (Figure 5D). These findings were also confirmed by the increase in cell viability of *ATG5-*silenced cells under DHEA treatment (Figure 5E). Altogether, these results imply that PERK UPR signaling and autophagy are required for DHEA-induced apoptosis with or without p53 function.

**FIGURE 6:**
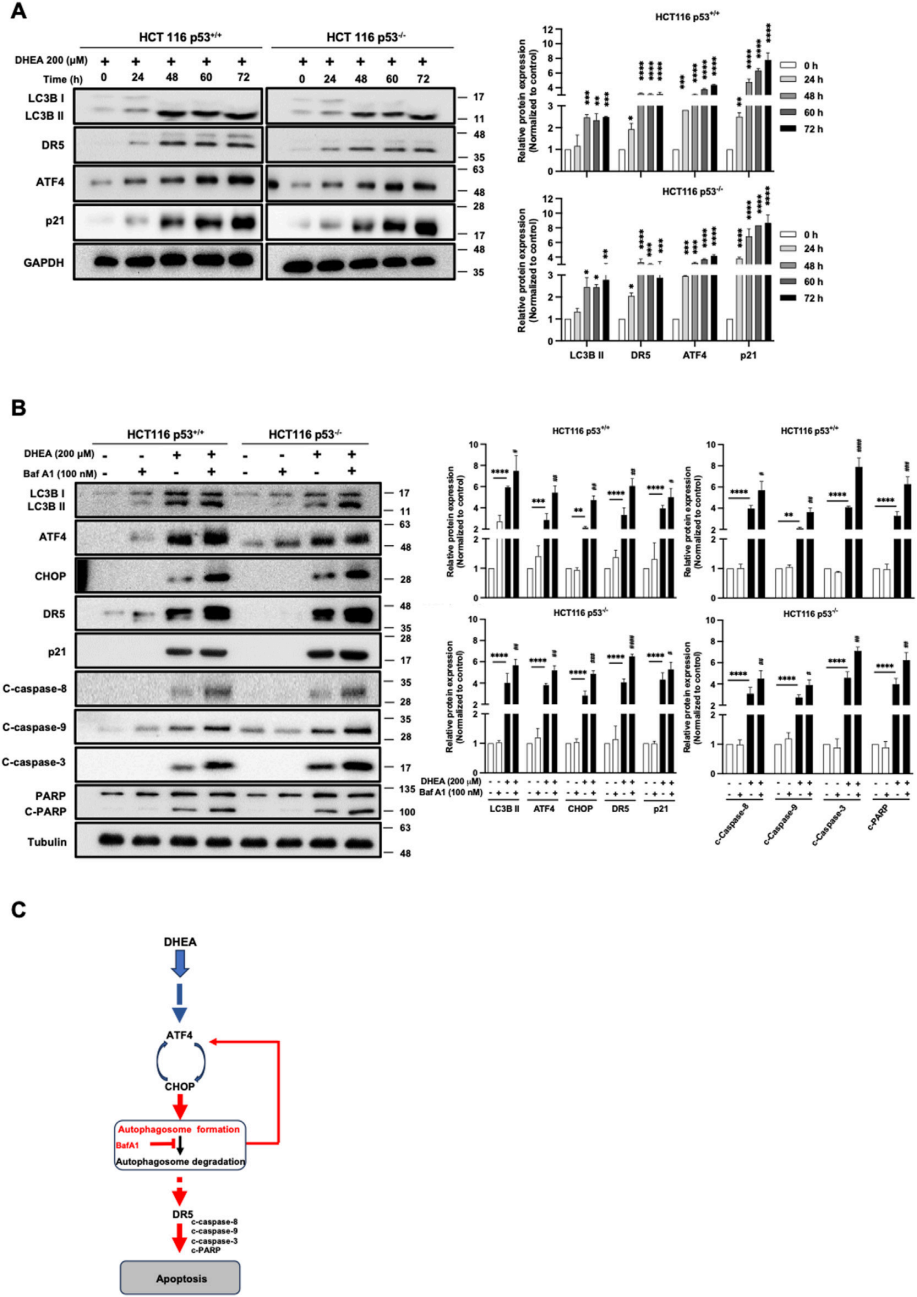
DHEA-induced autophagy degradation machinery leads to cell apoptosis in a p53-independent manner. **(A)** Western blot data of two HCT116 p53^+/+^ and p53^−/−^ cell lines were monitored from 0 to 72 h of LC3B, DR5, ATF4, and p21 protein levels using specific antibodies. **(B)** HCT116 p53^+/+^ and p53^−/−^ cell lines were treated with the fusion blocker bafilomycin A1 (BafA1, 100 nM) in the presence or absence of DHEA (200 µM) for 48 h. Western blot analysis was conducted to evaluate the levels of LC3B, ATF4, CHOP, p21, and apoptotic markers (DR5, caspase 8, 9, 3, and PARP) using specific antibodies. GAPDH and tubulin were used as the internal controls. Statistical data were collected from three independent experiments comparing the control and DHEA treatment groups. *p < 0.05; **p < 0.01; ***p < 0.001; ****p < 0.0001; and by comparing the control group with BafA1 treatment under DHEA treatment with ^#^ *p* < 0.05; ^##^ *p* < 0.01; ^##^ *p* < 0.01; ^####^ *p* < 0.0001*.* **(C)** Inhibition of the autophagy degradation by BafA1 enhanced DHEA-induced apoptosis, possibly through a feedback loop upregulation of the ATF4-CHOP axis and/or DR5 (in red color).

Moreover, to clarify how DHEA could affect autophagy progression, the level of LC3B II protein was measured from 0 h to 72 h in two HCT116 cell lines after DHEA (200 μM) treatment. Our data showed a steady increase of LC3B II level and coincided with levels of DR5, ATF4, and p21 from 0 h to 72 h p.t. (Figure 6A). To further clarify whether autophagy degradation machinery might participate in DHEA-induced autophagy progression. HCT116 p53^+/+^ and HCT116 p53^−/−^ cells were treated with the fusion blocker of autophagosome and lysosome bafilomycin A1 (BafA1, 100 nM) to block autophagy degradation in the presence or absence of DHEA (200 μM) for 48 h. The data showed that BafA1 effectively blocked autophagy degradation and caused the accumulation of a group of proteins, including LC3B II, ATF4 and CHOP (PERK pathway), p21, as well as apoptosis proteins, including DR5, cleaved caspase 8, 9, and 3, and cleaved PARP, in the cells with DHEA (200 μM) treatment compared to the cells with only BafA1 or DHEA treatment (Figure 6B). This result indicates that autophagy degradation machinery is involved in the regulation of ATF4, CHOP, and p21, as well as DR5 expression in the two DHEA-treated CRC cell lines, and p53 function is dispensable in this event. Taken altogether, these findings suggest that autophagy is a pivotal mediator of DHEA to exert the caspase-dependent cytotoxicity in CRC cells, and this effect does not require p53 involvement in the CRC cells (Figure 6C).

### DHEA suppresses tumor formation *in vivo*

**FIGURE 7:**
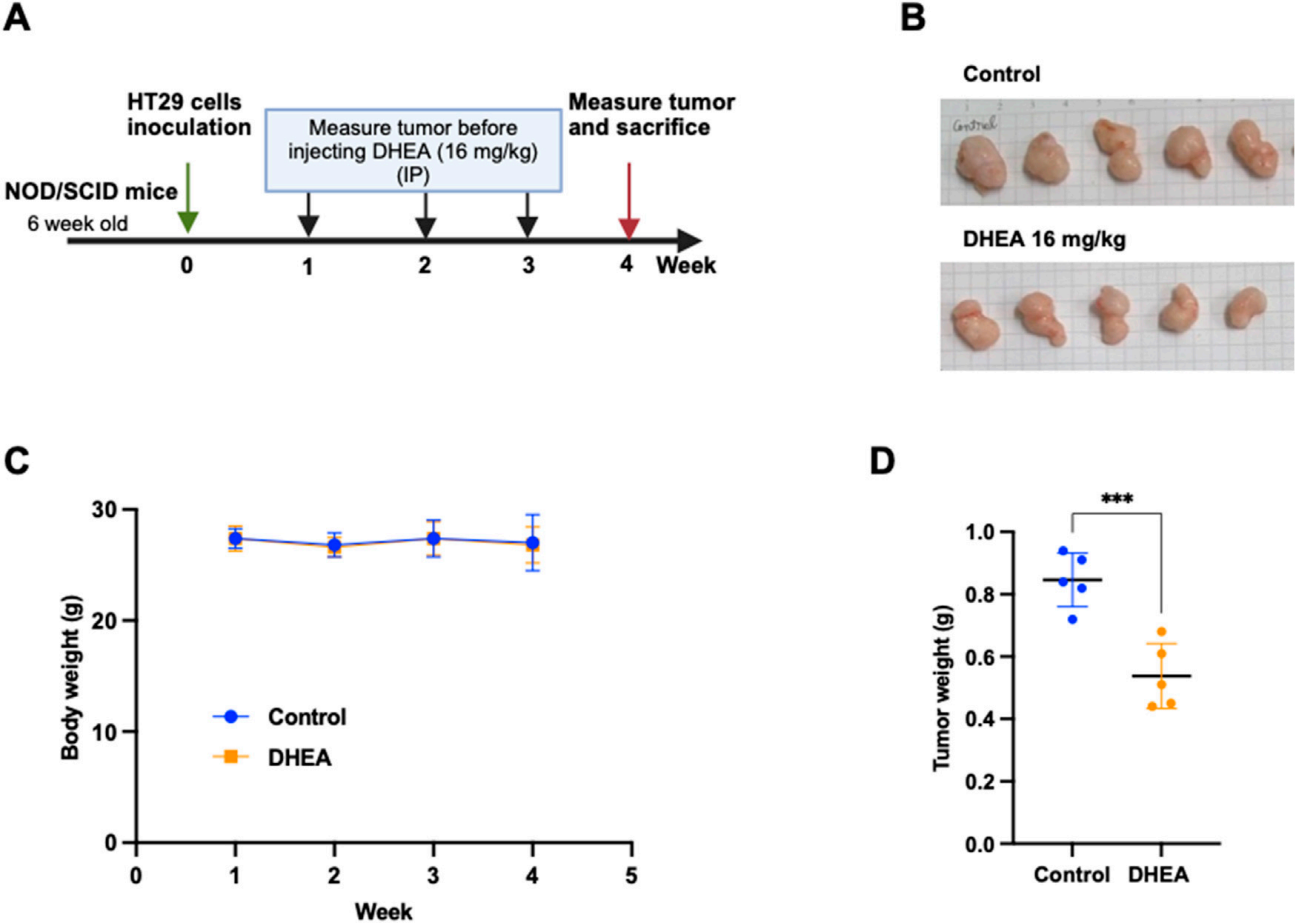
DHEA suppresses tumor formation of CRC cells in a xenograft mouse model. **(A)** The schedule of DHEA treatment on 6-week-old NOD/SCID mice. Briefly, HT29 cells were inoculated by subcutaneous injection and waited for tumor formation in 1 week (week 0). DHEA (16 mg/kg) was intraperitoneally injected into the mice three times (Wednesday, Thursday, and Friday) per week for 3 weeks (from weeks 1–3). The control group was treated with the same volume of DMSO. Body weight was measured once a week before DHEA injection. After 4 weeks, tumors were collected and measured. **(B)** Image of tumors of control and DHEA group after sacrificing at week 4 (n = 5). **(C)** Body weight of mice from week 1 to week 4 in untreated and treated mice. **(D)** is tumor weight at week 4 of the control and DHEA groups. Error bars present ± SD. Statistical analysis was obtained using a *t*-test with ***p < 0.001.

To validate the inhibitory effect of DHEA on tumor formation, CRC HT29 cells were inoculated subcutaneously into a xenograft mouse model. The procedure of treatment is described in Figure 7A. After DHEA treatment for 4 weeks, tumor weight was significantly reduced compared to the control group (Figures 7B,D). In contrast, the body weight of the mice was not changed (Figure 7C), indicating minor side effects of DHEA treatment. Taken together, DHEA could suppress tumor formation *in vivo* and may become a potential anti-cancer candidate for CRC treatment.

## Discussion

DHEA is a natural precursor hormone for androsterone and testosterone (Prough et al., 2016). Numerous studies have highlighted the repurposing potential of DHEA in the treatment of various cancers (Jiang et al., 2005; Li et al., 2022; Schulz et al., 1992; Vegliante et al., 2016; Yang et al., 2016). However, the relationship between p53 status and the inhibitory effect of DHEA remains unknown. Herein, we investigated the cytotoxicity of DHEA on various CRC cell lines harboring wild-type or mutant type of p53 gene to understand their relationship further. Our findings clarified 1) DHEA induces ER stress via PERK/eIF2α/ATF4/CHOP signaling pathway (Figure 3A); 2) p21 and autophagy are downstream of the PERK-ATF4-CHOP signaling pathway, and autophagy participates in DHEA-induced apoptosis (Figures 4–6); 3i) The inhibitory effect of DHEA is independent of p53 regulation (Figures 1–6).

The mechanisms of DHEA in suppressing cancer cell proliferation have been reported. For example, deletion of endogenous mevalonate or blockage of the protein isoprenylation or decreased PI3K/Akt pathway led to arrest G0/G1 phase of the cell cycle; however, there is no apoptotic effect reported at 24 h of DHEA treatment in HT29 CRC cell (Armaghany et al., 2012; Schulz et al., 1992; Jiang et al., 2005). DHEA has also been shown to induce apoptotic cell death in MCF7 cells by regulating transcriptional genes involved in p53 signaling pathway (Yang et al., 2016) and triggering autophagy-associated cell death in HepG2 cells (Vegliante et al., 2016). In our study, we demonstrated that DHEA not only inhibited G1 phase of cell cycle but also induced apoptosis through the activation of PERK-ATF4-CHOP axis under ER stress induction at 48 h after DHEA treatment in both wild-type and mutant p53 CRC cells (Figures 2–6).

Furthermore, numerous studies reported that ER stress triggered PERK-ATF4 signaling pathway may activate either survival or apoptotic response (Rozpedek et al., 2016). Activation of PERK may increase phosphorylation of eIF2α at serine 51 to enhance proteasomal degradation of Cyclin D1-a regulator of G1/S transition (Raven et al., 2008; Sherr and Roberts, 2004), or upregulate p21 mRNA – a G1 phase inhibitor by activated ATF4 independent of p53 regulation (Inoue et al., 2017), which prevents protein synthesis and cell proliferation. Similarly, our data showed the downregulation of Cyclin D1 and upregulation of p21 to inhibit cell proliferation in both wild-type and null p53 CRC cells (Figure 2C). Besides cell cycle arrest, activation of the eIF2α/ATF4 pathway may also upregulate autophagy-related genes to eliminate the non-necessary or overloaded failed proteins in the ER lumen (B'Chir et al., 2013). Specifically, *p62/SQSTM1, Nbr1,* and *ATG7* genes are upregulated by the binding of ATF4-CHOP complex to Amino Acid Response Element (AARE), and ATF4 activation can induce *ATG16/1, Map1lc3b, ATG12, ATG3, Becn1* and *Gabarapl2* genes. Moreover, CHOP can upregulate *ATG10, Gabarap,* and *ATG5* genes (B'Chir et al., 2013). Our data showed that silencing either *ATF4* or CHOP resulted in a significant downregulation of LC3B II induced by DHEA (Figure 4C), providing further evidence of the cooperative role of ATF4-CHOP in the regulation of autophagy. The transition between the survival and the pro-apoptotic signal depends on the duration of stress (Rozpedek et al., 2016). A prolonged G1 arrest has been reported to cause severe DNA damage due to the unrecovered DNA replication process, which leads to lasting proliferative suppression and effectively sensitizing chemo-drugs in combination therapies (Crozier et al., 2022). Moreover, persistent ER stress also leads to prolonged autophagy and consequently triggers cell death (Kwon et al., 2023). Similarly, our data showed that DHEA triggered a steady increase of ER stress to 48 h p.i (Figure 3B) accompanied by a prolonged induction of autophagy (Figure 6A). Additionally, Vegliante et al, (2016) reported that DHEA triggered autophagy-associated cell death by upregulating JNK signaling-mediated p62/SQSTM1 expression in HepG2 cells (Vegliante et al., 2016). Furthermore, Zhou et al, (2019) reported that Chrysanthemulide A blockage of the fusion of the autophagosome and the lysosome transcriptionally upregulates DR5 protein in both intracellular and membrane compartments of human osteosarcoma cells, leading to apoptosis through caspase 8 activation, and silencing either *LC3B* or *ATG5* with siRNA inhibited autophagosome formation and downregulated DR5 expression (Zhuo et al., 2019). Consistently, our study demonstrates that the DR5 protein level was regulated by ATF4-CHOP-mediated degradative autophagy under the treatment of DHEA (Figures 4D, 6B). Moreover, ATF4 activates CHOP to induce apoptosis by transactivating BIM, suppressing BCL-2, and upregulating PUMA and BAX/BAK (Vandewynckel et al., 2013; Rozpedek et al., 2016). CHOP also induces DR5 by increasing the activity of the DR5 gene promoter (Chen et al., 2017). On the other hand, PUMA induces mitochondrial autophagy, leading to apoptosis dependent on BAX/BAK upregulation (Thorburn et al., 2014; Yee et al., 2009). Our data also showed that DHEA regulated both DR5 and PUMA through autophagy function (Figure 4D), which has not been reported in other studies. Therefore, the relationship among DR5, PUMA, and autophagy warrants further exploration.

**FIGURE 8:**
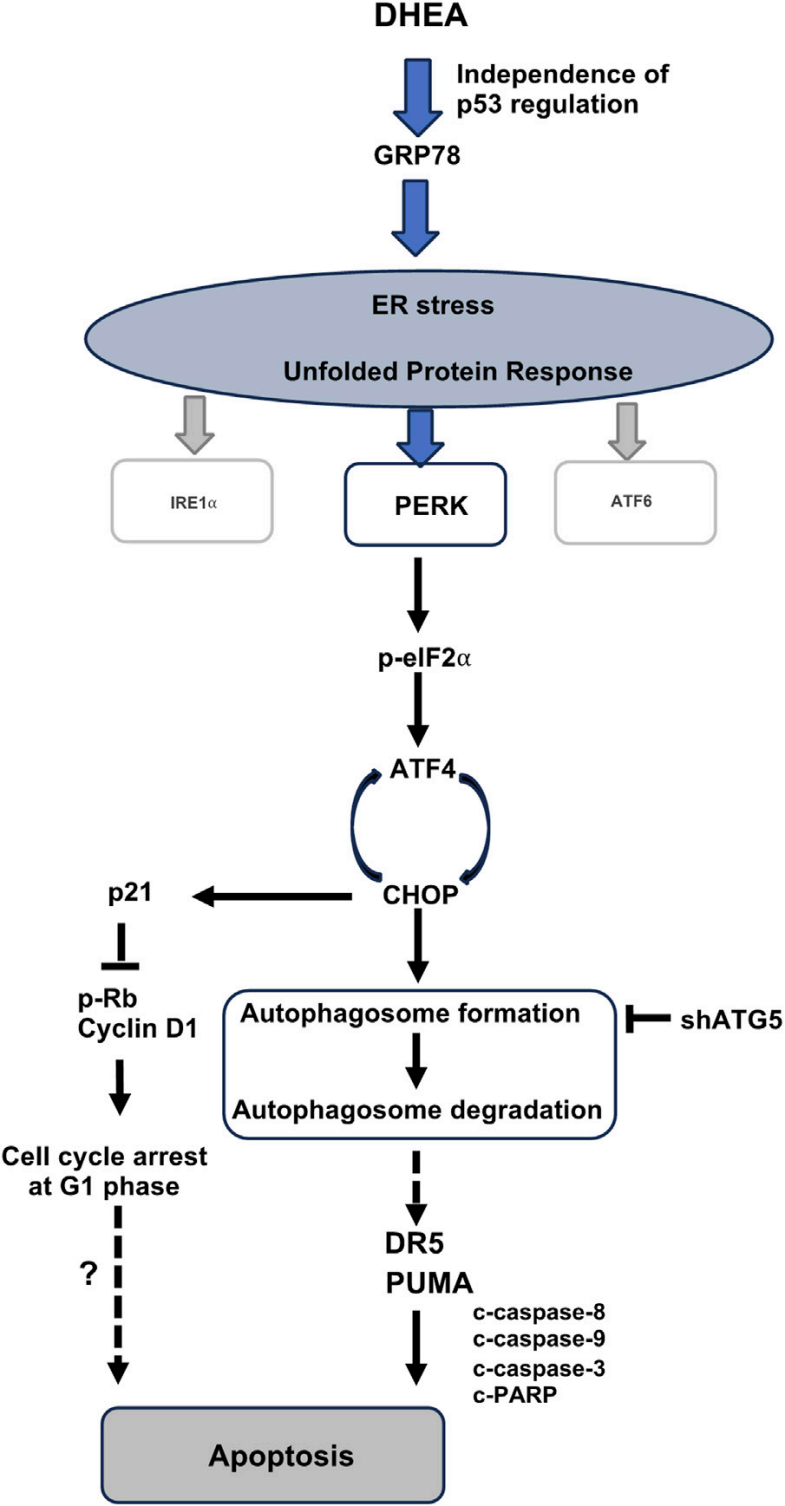
A schematic diagram depicts the molecular mechanism of DHEA suppressing CRC progression. DHEA induces ER stress and the downstream PERK/p-eIF2⍺/ATF4/CHOP UPR signaling in CRC cells, independence of p53 regulation. Subsequent interaction of activated ATF4-CHOP leads to activated autophagy (the box presents two steps in the autophagy process, including autophagosome formation and autophagosome degradation by the fusion of autophagosome and lysosome), which is a critical mediator for DHEA-induced apoptotic cell death. DR5 and PUMA upregulation through autophagy induction triggers apoptosis after DHEA treatment (Figure 4D). Besides apoptosis, ATF4 activation also upregulates the p21 protein level (Figure 3D) as a result of the induction of G1 arrest. However, whether the arrest in G1 leads to apoptosis under DHEA treatment warrants further exploration.

In summary, we are the first to reveal that DHEA triggers ER stress-PERK UPR pathway-mediated autophagy followed by apoptotic death of CRC cells (Figure 8). Notably, DHEA exerts its anticancer function by arresting cell cycle at G1 phase, suppressing cell proliferation, colony formation, and tumor formation in a p53-independent manner. Our findings provide valuable insights for tailoring treatment regimens and predicting DHEA’s efficacy in further application for patients with different p53 status.

## Data Availability

The original contributions presented in the study are included in the article/Supplementary Material, further inquiries can be directed to the corresponding authors.

## Abbreviations

APC, Adenomatous Polyposis Coli; ATF4, Activating transcription factor 4; ATF6, Activating transcription factor 6; ATG5, Autophagy-related protein 5; BafA1, Bafilomycin A1; CCK-8, Cell Counting kit – 8; CHOP, C/EBP homologous protein; CRC, Colorectal Cancer; DHEA, Dehydroepiandrosterone; DR4 and DR5, Death receptor 4 and 5; eIF2α, Eukaryotic translation initiation factor 2A; ER, Endoplasmic Reticulum; IRE1α, Inositol-requiring kinase/endoribonuclease1; KRAS, Kirsten rat sarcoma virus; PERK, Protein kinase R-like Endoplasmic reticulum kinase; PUMA, P53 upregulated modulator of apoptosis; SRB, Sulforhodamine B; 4-PBA, 4-phenyl-butyric acid; UPR, Unfolded Protein Response.
