# Supplementary material for: Dehydroepiandrosterone suppresses human colorectal cancer progression through ER stress-mediated autophagy and apoptosis in a p53-independent manner
Source: Front Pharmacol. 2024 Oct 2;15:1464647. doi: 10.3389/fphar.2024.1464647 (PMC11487585; doi:10.3389/fphar.2024.1464647)
Supplement: Supplementary file 1 [file DataSheet1.docx]

**Table S1** Target gene sequences of lentiviral shRNA

| Gene (^a^shRNA) | Sequence of target gene | ^b^MOI |
| --- | --- | --- |
| *DDIT3 (CHOP)* | ^5^CTGCACCAAGCATGAACAATT^3^ | 2 |
| *ATF4* | ^5^CCACTCCAGATCATTCCTTTA^3^ | 2 |
| *ATG5* | ^5^CCTTTCATTCAGAAGCTGTTT^3^ | 2 |
| GFP or Luciferase vector | ^5^ACAACAGCCACAACGTCTATA^3^ | 2 |

^a^lentiviral shRNA

^b^MOI: multiplicity of infection

**Table S2** Antibodies used in this study

| No | Antibody | Cat. No. | Company | Host | Dilution |
| --- | --- | --- | --- | --- | --- |
| 1 | Rb | 9313S | Cell signaling | Rabbit | 1:1000 |
| 2 | p-Rb^ser795^ | 9307 | Cell signaling | Rabbit | 1:1000 |
| 3 | Cyclin D1 | 55506 | Cell signaling | Rabbit | 1:1000 |
| 4 | p21 | 2947 | Cell signaling | Rabbit | 1:1000 |
| 5 | p53 | Sc-126 | Santacruz | Mouse | 1: 250 |
| 6 | Puma | 4976 | Cell signaling | Rabbit | 1:1000 |
| 7 | Bak | 3814 | Cell signaling | Rabbit | 1:1000 |
| 8 | Bcl-2 | 3498 | Cell signaling | Rabbit | 1:1000 |
| 9 | c-caspase 7 | 9491S | Cell signaling | Rabbit | 1:1000 |
| 10 | c-caspase 8 | 13423-1-AP | Proteintech | Rabbit | 1:1000 |
| 11 | c-caspase 9 | 10380-1AP | Proteintech | Rabbit | 1:1000 |
| 12 | c-caspase 3 | 9661S | Cell signaling | Rabbit | 1:1000 |
| 13 | PARP | 9542S | Cell signaling | Rabbit | 1:1000 |
| 14 | GRP78/BiP | 3177P | Cell signaling | Rabbit | 1:1000 |
| 15 | IRE1α | 3294P | Cell signaling | Rabbit | 1:1000 |
| 16 | ATF6 | 65880 | Cell signaling | Rabbit | 1:1000 |
| 17 | PERK | 5683 | Cell signaling | Rabbit | 1:1000 |
| 18 | p-PERK | Ab192591 | Abcam | Rabbit | 1:1000 |
| 19 | eIF2α | 5234 | Cell signaling | Rabbit | 1:1000 |
| 20 | p- eIF2α | 3398 | Cell signaling | Rabbit | 1:1000 |
| 21 | ATF4 | 11815 | Cell signaling | Rabbit | 1:1000 |
| 22 | CHOP | 5554 | Cell signaling | Rabbit | 1:1000 |
| 23 | DR5 | 8074 | Cell signaling | Rabbit | 1:1000 |
| 24 | p62 | Ab56416 | Abcam | Mouse | 1:1000 |
| 25 | LC3B | 3868 | Cell signaling | Rabbit | 1:1000 |
| 26 | Atg5 | 2630S | Cell signaling | Rabbit | 1:1000 |
| 27 | G6PD | 8866 | Cell signaling | Rabbit | 1:1000 |
| 28 | GAPDH | Sc-32233 | Santacruz | Mouse | 1:1000 |
| 29 | α-Tubulin | A6830 | ABclonal | Rabbit | 1:1000 |

**Table S3** IC_50_ values of DHEA treated on CRC cell lines harboring different p53 genes at 24, 48, and 72 h

| Cell lines | p53 status | ^a^IC_50_ of DHEA (μM) | | |
| --- | --- | --- | --- | --- |
|  |  | **24 h** | **48 h** | **72 h** |
| HCT116 p53^+/+^ | ^b^Wild type | 363.2 ± 20.7 | 137.6 ± 13.2 | 91.5 ± 7.8 |
| RKO | Wild type | 311.5 ± 3.8 | 128.0 ± 11.7 | 102.7 ± 10.7 |
| HT29 | ^c^Mutant (R273H) | > 400 | 216.1 ± 41.9 | 98.2 ± 11.5 |
| LS1034 | Mutant (G245S) | > 400 | 204.7 ± 36.1 | 103.9 ± 11.4 |
| HCT116 p53^-/-^ | ^d^Null | > 400 | 217.3 ± 20.3 | 138.6 ± 9.5 |
| ^a^ IC_50_: 50% inhibitory concentration of DHEA on CRC cell number; IC_50_ values were calculated based on percentages of growth inhibition determined by the SRB assay. Data were performed by mean ± Standard Error of Mean (SEM) from three independent experiments (*N=3*).  ^b^Wild type: wild type p53 gene  ^c^Mutant: mutant p53 gene (R273H) or (G245S)  ^d^Null: without p53 gene (p53^-/-^) | | | | |

**
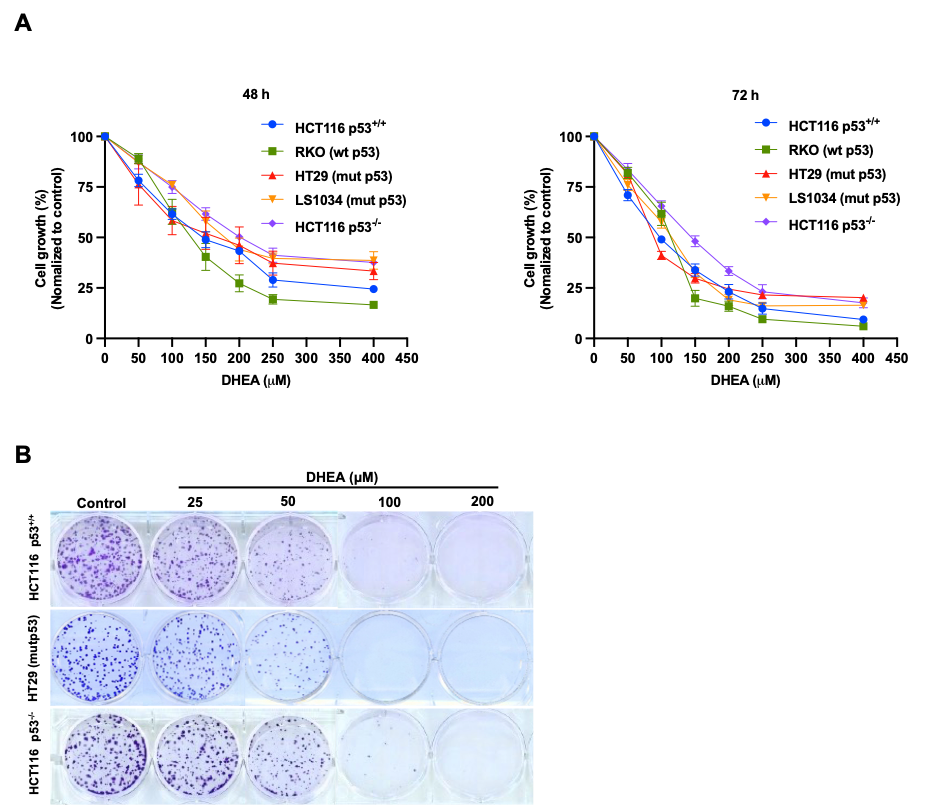
**

**Figure S1.** DHEA suppresses human CRC cells' growth and colony formation regardless of p53 status; (**A**) Percentage of cell growth of 5 CRC cells after DHEA treatment at a dose range (0 to 400 µM) at 48 and 72 h. The cell growth was determined by a sulforhodamine B (SRB) assay, and the optical density (OD) wavelength was measured at 510 nm. (**B**) Images of colony formation inhibition of p53^+/+^ HCT116, HT29, and p53^-/-^ HCT116 cells after DHEA treatment at 0, 25, 50, 100, and 200 µM for 12 days. Then, colonies were stained by crystal violet 0.5% and captured by a scanner.

**
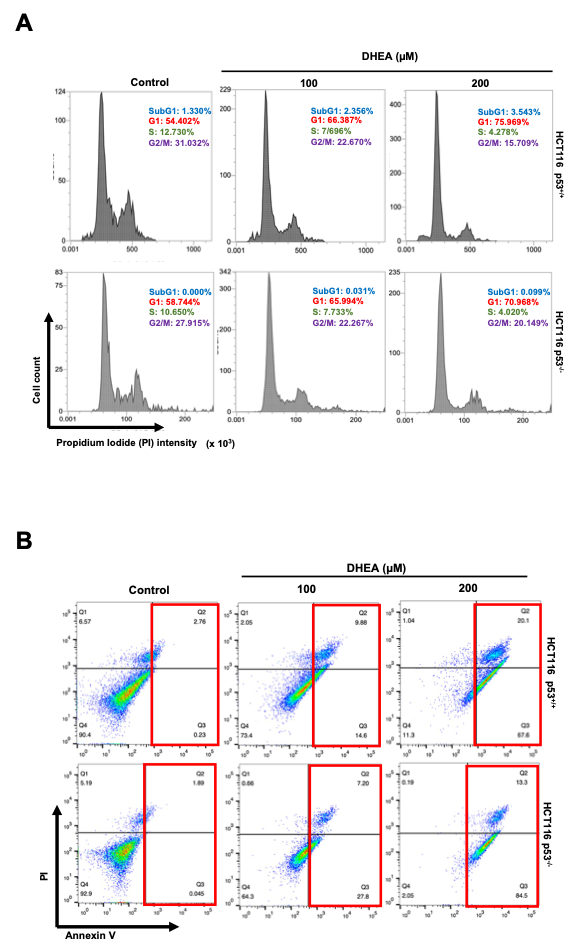
**

**Figure S2.** DHEA suppresses the CRC cell viability by inducing G1 phase arrest and apoptosis in a p53-independent manner; (**A**) Histogram of percentage of cell population at each phase of the cell cycle of p53^+/+^ and p53^-/-^ HCT116 after DHEA treatment for 48 h, analyzed by flow cytometry. (**B**) The percentage of apoptotic cells was stained with only Annexin V or combined with propidium iodide (PI) (red rectangle) after DHEA (0, 100, and 200 μM) treatment at 48 h and determined by flow cytometry.

**
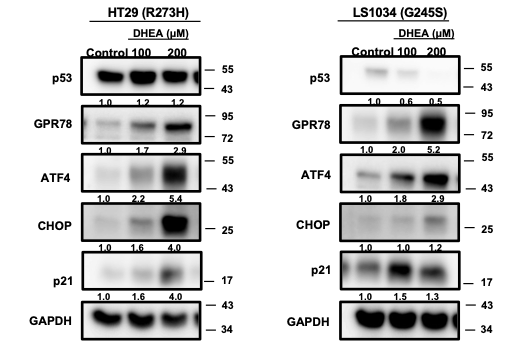
**

**Figure S3.** DHEA induces ER stress through the PERK/eIF2/ATF4/CHOP signaling pathway in a p53-independent manner; Western blotting data of specific antibodies such as p53, ER stress marker (GPR78 or BiP), PERK signaling pathway (ATF4 and CHOP) were identified after DHEA treatment at 0, 100, 200 µM in mutant p53 HT29 (R273H) and LS1034 (G245S) cells for 24 h. GADPH was used as an internal control. Images were captured by Multigel 21. The intensity of bands was quantified by MultiGauge 3.0 software.

**
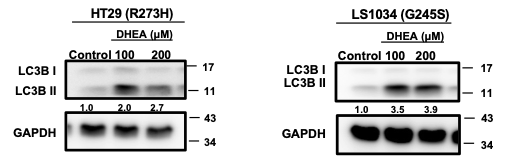
**

**Figure S4.** DHEA-triggered PERK/ATF4/CHOP signaling pathway induces autophagic activity in a p53-independent manner; Western blotting data of LC3BII, an autophagy marker, was determined after 24 h post-DHEA treatment at 0, 100, and 200 µM in HT29 and LS1034 cells. GADPH was used as an internal control. Images were captured by Multigel 21. The intensity of bands was quantified by MultiGauge 3.0 software.


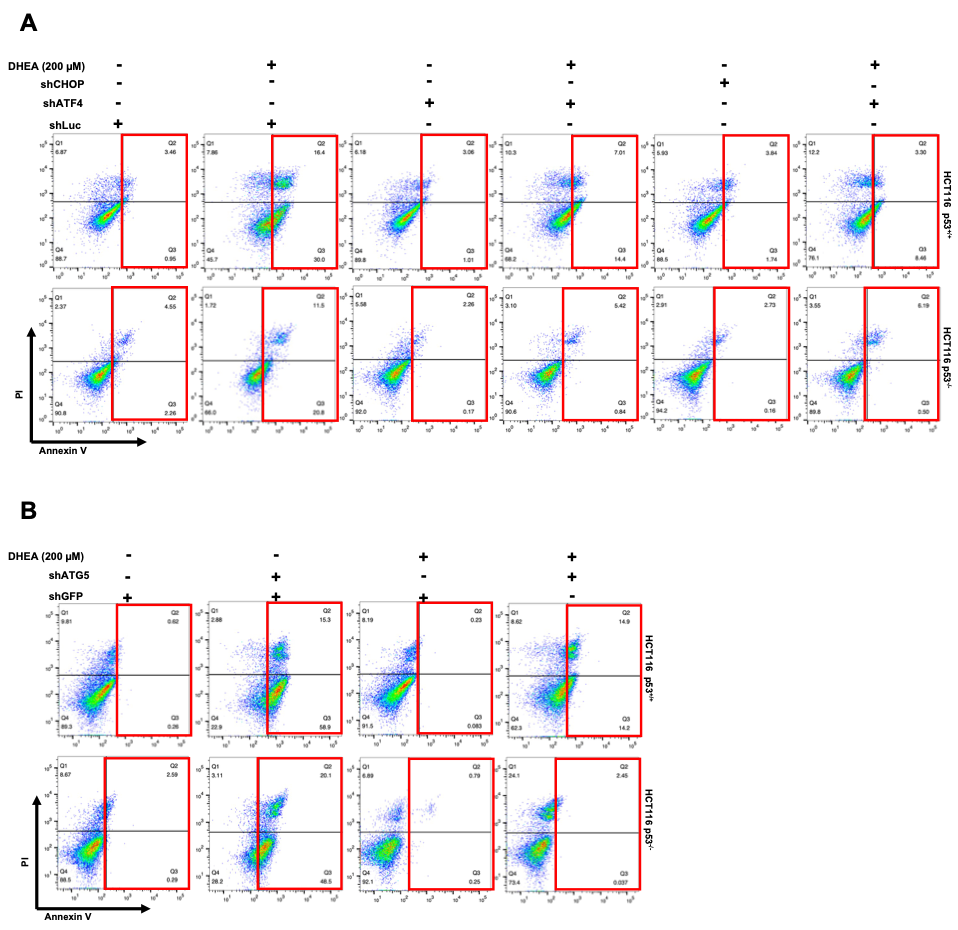


**Figure S5.** DHEA-induced autophagy degradation machinery leads to cell apoptosis in a p53-independent manner. The percentage of apoptotic cells (in red rectangle) was stained with only Annexin V or combined with Propidium iodide (PI) (red area) after DHEA treatment at 48 h and determined by flow cytometry. (**A**) Two HCT116 cells were silenced by lentiviral plasmid shLuc (control), shATF4, and shCHOP, and (**B**) cells were knocked down by shGFP (control) and shATG5.
